# Btbd7 contributes to reduced E-cadherin expression and predicts poor prognosis in non-small cell lung cancer

**DOI:** 10.1186/1471-2407-14-704

**Published:** 2014-09-24

**Authors:** Chuifeng Fan, Yuan Miao, Xiupeng Zhang, Di Liu, Guiyang Jiang, Xuyong Lin, Qiang Han, Lan Luan, Zhonghai Xu, Enhua Wang

**Affiliations:** Department of Pathology, First Affiliated Hospital and College of Basic Medical Sciences of China Medical University, Shenyang, 110001 China; Institute of Pathology and Pathophysiology, China Medical University, Shenyang, 110001 China

**Keywords:** Btbd7, E-cadherin, NSCLC, Prognosis

## Abstract

**Background:**

Disorders of cell adhesion are critical steps in cancer progression in which varieties of markers including cadherins are involved in.Btbd7 was found to inhibit E-cadherin expression in MDCK cells and play important roles during branching morphogenesis of embryonic salivary glands and lungs. However its function in malignant tumors is largely unknown. The aim of this study is to investigate the clinicopathological significance and possible function of Btbd7 in non-small cell lung cancer.

**Methods:**

Immunohistochemistry and Western blotting were used to investigate Btbd7 expression in non-small cell lung cancer and lung tissues. The clinicopathological association and the overall survival was analyzed. In vitro experiments were performed using siRNA to investigate the function of Btbd7 in lung cancer cells.

**Results:**

Btbd7 expression was elevated in non-small cell lung cancer tissues compared to normal lung tissues. Increased Btbd7 expression was significantly associated with lymph node metastasis, reduced E-cadherin expression and patients’ poor clinical outcome. Downregulation of Btbd7 expression in lung cancer cells by siRNA significantly inhibits cancer cell invasion and effectively restores E-cadherin expression in cancer cell membrane.

**Conclusions:**

Btbd7 contributes to reduced expression of E-cadherin and may be a promising cancer marker in non-small cell lung cancer.

**Electronic supplementary material:**

The online version of this article (doi:10.1186/1471-2407-14-704) contains supplementary material, which is available to authorized users.

## Background

The occurrence of invasion and metastasis of lung cancer cells are often the main difficulties in the treatment of this tumor. Our previous studies have indicated that abnormalities in cancer cell adhesion at the level of the E-cadherin complex are involved in the invasion and metastasis of lung cancer [1, 2]. E-cadherin is one of the key cadherins and plays major roles in the establishment and maintenance of intercellular adhesion, cell polarity and tissue architecture [3]. Abnormalities in expression, cellular distribution and function of E-cadherin are frequently indicated in development including invasiveness, lymph node metastasis and distance metastasis in a variety of human malignancies [4, 5]. Various factors have been found to regulate expression and function of E-cadherin in malignant tumors and implicated in cancer progression [6–14]. Recently Btbd7 (BTB (POZ) domain containing 7), a BTB (POZ) domain containing protein, was found to play important roles in the development of salivary glands and lungs through regulating E-cadherin [15]. Many organs form by branching of epithelia through the formation of clefts and buds during embryonic development. The authors identified Btbd7 as a dynamic regulator of branching morphogenesis through its highly focal expression leading to local regulation of E-cadherin and epithelial cell motility [15]. Btbd7 protein contains 1130 amino acids with two putative BTB/POZ domains. The protein family containing BTB domains are evolutionarily conserved from Drosophila to mammals [15]. The BTB domain is a protein-protein interaction motif that was first identified as a sequence motif in genes of DNA virus [16]. The functions of BTB-containing proteins well known now are mainly transcriptional regulation and protein degradation [16]. Btbd7 was originally indentified as a regulatory gene that promotes epithelial tissue remodeling and formation of branched organs [15]. However, it is still not clear whether Btbd7 is also involved in the process of invasion and metastasis of lung cancer cells. So far, expression of Btbd7 and its function in malignant tumors are largely unknown. The purpose of this study is to investigate Btbd7 expression and its clinicopathological significance in non-small cell lung cancer (NSCLC). In addition, we used specific siRNA to downregulate Btbd7 expression to investigate its possible function to impact E-cadherin expression and invasion ability in lung cancer cells in vitro.

## Methods

### Tissue samples

Tumor specimens including NSCLC tissues and paired non-tumor portion (with >5 cm distance from the primary tumor’s edge) from 130 patients with NSCLC were obtained between 2003 and 2009 following surgical resection at the First Affiliated Hospital of China Medical University. Of the 130 lung cancer cases, 86 contained complete follow-up data. None of the patients had received radiotherapy, chemotherapy, or immunotherapy prior to tumor excision. Of the patients, 87 are male and 43 are female, creating a 2.02:1 ratio of male to female. Patients’ ages at the time of surgery ranged from 33 to 80, with an average age of 58.8 years old. The tumors were classified according to the TNM stage revised by the International Union Against Cancer (UICC) [17]. All specimens were re-evaluated for diagnosis following the criteria for classification of lung cancer by the World Health Organization (WHO) [18] and the revised edition for lung carcinoma in 2010 [19], and 62 squamous cell carcinomas (SCCs) and 68 adenocarcinomas were confirmed. Relevant clinical data from the patients included in the study can be seen in Additional file 1. This study was approved by the Institutional Review Board of China Medical University and conducted under the regulations of it. Informed consent was obtained from all enrolled patients prior to surgery.

### Immunohistochemistry

Formalin-fixed, paraffin-embedded specimens were cut into 4 μm-thick sequential sections. The sections were dewaxed in xylene and rehydrated stepwise in descending ethanol series. Endogenous peroxidase activity and non-specific binding were blocked with 3% H_2_O_2_ and non-immune sera, respectively. The sections were then incubated with primary goat anti-human polyclonal antibody Btbd7 (ab121006, abcam, HK; dilution 1:50), mouse anti-human monoclonal antibody E-cadherin (ab1416, abcam, HK; dilution 1:100) and N-cadherin (ab98952, abcam, HK; dilution 1:100) overnight at 4°C. Thereafter, the catalyzed signal amplification system (Maixin Biotechnology, Fuzhou, Fujian, China) was used for staining of these proteins according to the manufacturer’s instructions. The antibodies were detected by a standard avidin-biotin complex method with biotinylated secondary antibodies (Maixin) and an avidin-biotin complex (Maixin), and developed with diaminobenzidine. Counterstaining was done lightly with hematoxylin, and the sections were dehydrated in alcohol before mounting.

The sections were assessed by at least three observers who had no knowledge of the patients’ clinical status and independently from each other. Cases with discrepancies were jointly re-evaluated by the investigators, and a consensus was obtained. Scoring of IHC was based on two parameters: the proportion of immunopositive cells and their intensity of immunoreactivity. The proportion of immunopositive cells was categorized as follows: 0: <10%; 1: ≥10% to <25%; 2: ≥25% to <50%; 3: ≥50% to <75% and 4: ≥75%. The staining intensity was categorized by relative intensity as follows: 0: no positivity; 1: weak; 2: moderate and 3: strong. A final immunoreactivity score of each section was obtained by multiplying the two individual scores. To obtain final statistical results, a final score less than 2 was considered as negative, while scores of 2 or more were considered as positive. Membranous expression of E-cadherin showed no significant variations in intensity of staining, and the scoring system was applied based on the percentage of positive cells: 0: <10%; 1: ≥10% to <25%; 2: ≥25% to <50%; 3: ≥50% to <75%, and 4: ≥75%. Since the adjacent non-neoplastic bronchial epithelium had a score of 4 for membranous localization of Ecadherin, cases with scores <4 was defined as decreased Ecadherin expression, while cases with a score of 4 were defined as preserved E-cadherin expression.

### Immunofluorescent staining

Cells were fixed with 4% paraformaldehyde, followed by blocking with 1% BSA. They were then incubated with mouse anti-human monoclonal antibody E-cadherin (ab1416, abcam, HK; dilution 1:100) overnight at 4°C. The primary antibodies were followed by incubation with secondary antibodies conjugated to rhodamine. The nuclei were counterstained with DAPI. The cells were examined with an Olympus IX51 fluorescent microscope (Olympus, Tokyo, Japan), and images were captured with a CoolPIX 5400 camera (Nikon, Japan).

### Western blotting

Tissues and cells were lysed in 10 volumes (w/v) of lysis buffer. After centrifugation, the supernatant was collected and quantified. The same amount of total protein was separated by 10% SDS-PAGE and then transferred to a PVDF membrane. The membrane was then incubated overnight at 4°C with primary goat anti-human polyclonal antibody Btbd7 (ab121006, abcam, HK; dilution 1:500) and slug (sc-10436, Santa Cruz, USA, dilution 1:200), mouse anti-human monoclonal antibody E-cadherin (ab1416, abcam, HK; dilution 1:500), N-cadherin (ab98952, abcam, HK; dilution 1:500) and GADPH (ab8245, Abcam, HK; 1:1000) and mouse anti-human polyclonal antibody β-actin (ab20272, Abcam, HK; 1:500). After incubation with the secondary antibody labeled with HRP at room temperature for 2 h, protein bands were visualized using enhanced chemiluminescence (ECL) and detected using the BioImaging System.

### Cell culture and transfection

Human bronchial epithelial cell HBE, and lung carcinoma cell lines including A549, NCI-H1299, NCI-H157, NCI-H460, LH7, LTE and SPC were cultured in RPMI 1640 tissue culture medium (Invitrogen, Carlsbad, CA, USA), containing 10% fetal calf serum (Invitrogen), 100 IU/mL penicillin (Sigma, St Louis, MO, USA), and 100 μg/mL streptomycin (Sigma) at 37°C in a humidified atmosphere (5% CO2, 95% air). HBE, A549, NCI-H1299, NCI-H460 and NCI-H157 were obtained from the American Type Culture Collection (Manassas, VA, USA). PG-LH7 (LH7) was a gift from Professor Jie Zheng from Bejing University. SPC-A-1 (SPC) and LTEP-A-2 (LTE) were obtained from the cell bank of Chinese Academy of Science. Btbd7 siRNA (sc-92326) and negative control siRNA (sc-37007) were purchased from Santa Cruz Biotechnology (Santa Cruz, CA, USA). For transient transfection, the cells were cultured in a 24-well plate for 24 h before the experiment. Then the cells were transfected with Lipofectamine 2000 (Invitrogen, Carlsbad, CA) according to the manufacturer’s instructions. Following the transfection, the cells were harvested at 24–48 hours to measure the protein levels.

### Matrigel invasion assay

The cells’ invasive abilities were examined using a 24-well Transwell with 8-μm pore polycarbonate membrane inserts (Corning, NY, USA) and matrigel (BD Bioscience) according to the manufacturer’s instructions. 20 ul Matrigel (1:3 dilution) was added to each insert. 100 ul cell suspension containing 3 × 10^5^ cells cells was transferred to the upper chamber and incubated for 36–48 h. The filters were stained with hematoxylin. Cells that appeared on the lower surface of the filter were counted in five random high-magnification microscope. Each experiment was done three times independently.

### Scratch wounding assay

Cell suspension is added in 6-well plates. After monolayer of adherent cells is formed draw two vertical lines using sterile 100 ul pipette tips then cells were washed 3 times with PBS to remove float cells from the mark. Mitomycin C (1 ug/ml) (Sigma-Aldrich UK) was added to the cells to inhibit cell proliferation 1 h before drawing the lines.Then the plates are moved to an incubator (5% CO2, 37°C). After incubation of 24-72 h, scratch width is observed under a stereomicroscope. Each experiment was done three times independently.

### Statistical analysis

SPSS statistical software package version 13.0 (SPSS Inc., Chicago, IL, USA) was used for all analyses. The Pearson’s Chi-Square test was used to analyze the relationship between Btbd7 expression and clinicopathological factors. The McNemar’s test was used to compare Btbd7 expression in normal lung tissues and lung cancer tissues. All data were expressed as means ± standard deviation (S.D.) for in vitro experiments performed at least 3 times. P-values of <0.05 were considered statistically significant.

## Results

### Quantification of Btbd7 expression in NSCLC and corresponding non-tumor lung tissues

**Figure 1:**
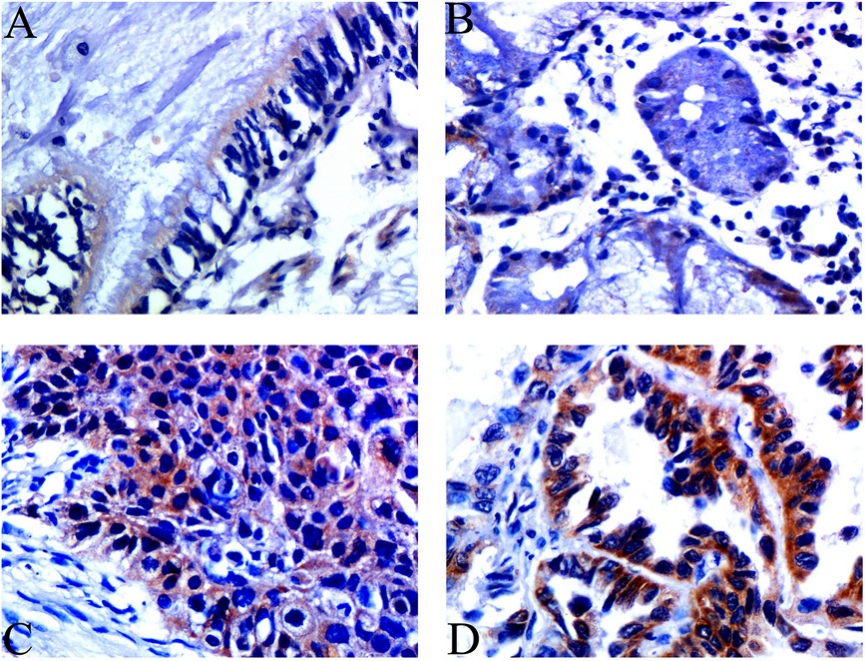
**Detection of Btbd7 expression using immunohistochemistry.** Btbd7 immunostaining was located mainly in cytoplasm. Btbd7 expression in bronchial epithelia **(A)** and submucosal glands **(B)** was commonly very weak or absent, while squamous carcinoma **(C)** and adenocarcinoma **(D)** cells often show stronger immunostaining of Btbd7 (×400).

**Figure 2:**
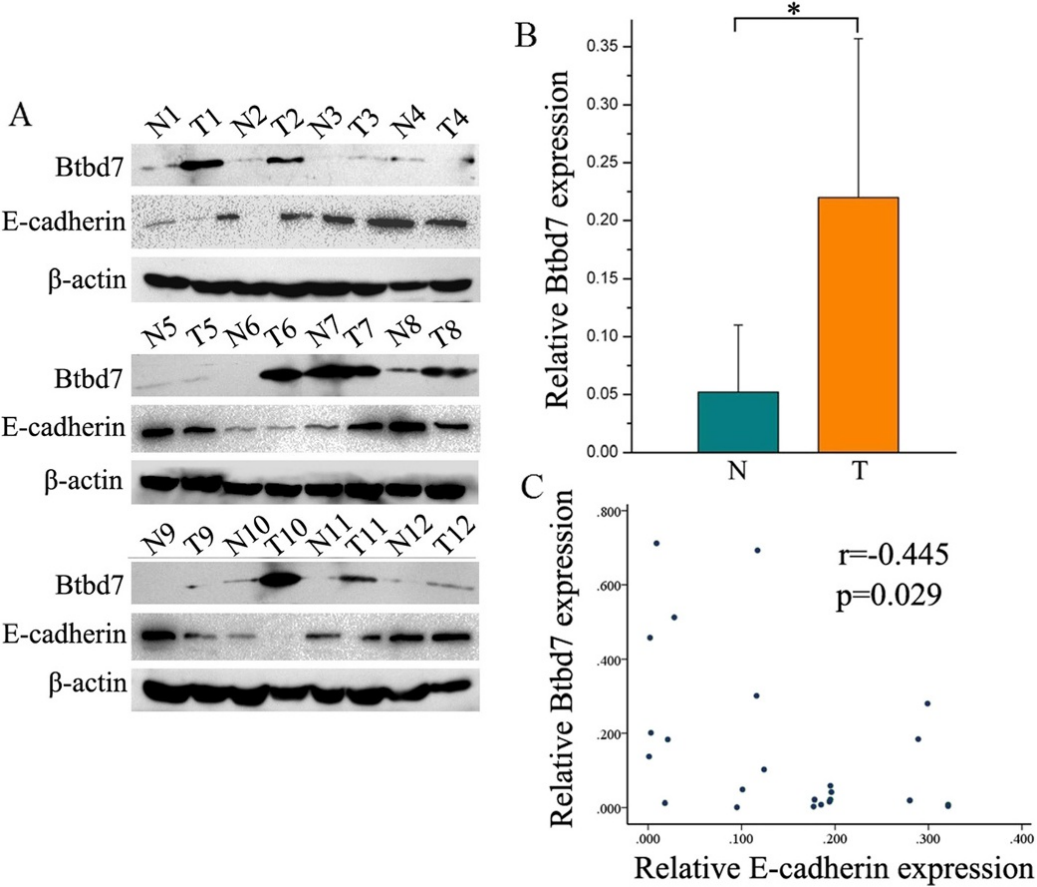
**Detection of Btbd7 expression using Western blotting. (A, B)** Western blotting study shows increased Btbd7 expression in cancer tissues (T) compared to corresponding non-tumor lung tissues (N) (* *p* < 0.05). **(A, C)** E-cadherin expression was negatively associated with Btbd7 expression in these tissues (r = −0.045, *p* < 0.05).

We performed immunohistochemistry (IHC) analysis and Western blotting study to investigate Btbd7 expression in NSCLC and corresponding non-tumor lung tissues. The IHC study shows Btbd7 expression is located mainly in cytoplasm (Figure 1). Normal bronchial epithelial cells and submucosal gland cells usually exhibit negative or weak staining (Figure 1, A, B), while the staining in cancer cells is often stronger (Figure 1, C, D). Total positive rate of Btbd7 expression in NSCLC was 51.5% (67/130) and higher than that in normal lung tissues (11.3%) (6/53) (p < 0.05). Western blotting study confirms increased expression of Btbd7 in NSCLC tissues compared to lung tissues (*p* < 0.05) (Figure 2,A,B). E-cadherin expression was negatively associated with Btbd7 expression in these tissues (r = −0.045, *p* < 0.05) (Figure 2, A, C).

### Association between Btbd7 expression and clinicopathological parameters in NSCLC

The relationship between Btbd7 expression and different clinicopathological factors in NSCLC is shown in Table 1. Increased Btbd7 expression in NSCLC was significantly associated with histological type, lymph node metastasis and TNM stages (*p* < 0.05) (Table 1). Higher Btbd7 expression was found in adenocarcinomas compared to squamous cell carcinomas (*p* < 0.05). Btbd7 expression was higher in cases with lymph node metastasis than those without lymph node metastasis (*p* < 0.05). Higher expression of Btbd7 was also found in cancer with advanced TNM stages (III + IV) (*p* < 0.05). There was no significant association between Btbd7 expression and the other clinicopathological factors in NSCLC.

**Table 1:** **Relationship between Btbd7 expression and clinicopathological factors in 130 patients with non-small cell lung cancer**

Variables	All patients	Btbd7 expression			
		Negative	Positive	*p* ^*^	*X* ^*2*^
**Total**	130	63	67		
**Age(y)**					
<55	47	20	27	0.310	1.029
≥55	83	43	40		
**Gender**					
Male	87	44	43	0.493	0.470
Female	43	19	24		
**Histological type**					
Squamous cell carcinoma	62	38	24	**0.005**	7.810
Adenocarcinoma	68	25	43		
**Grade**					
Well and moderate	93	47	46	0.453	0.564
Poor	37	16	21		
**TNM stage**					
Land II	72	41	31	**0.031**	4.650
III and IV	58	22	36		
**Lymph node metastasis**					
Yes	60	21	39	**0.004**	8.085
No	70	42	28		

*p values were obtained with the X^2^ test. The bold numbers were considered statistically significant.

### Btbd7 expression was associated with abnormal E-cadherin and N-cadherin expression in NSCLC

**Figure 3:**
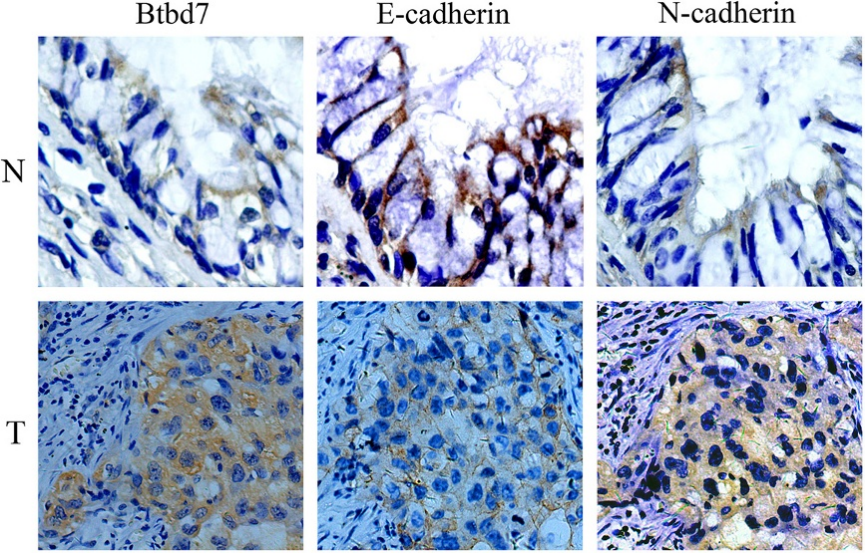
**Investigation of E-cadherin and N-cadherin expression in non-small cell lung cancer (T) and lung tissues (N) using immunohistochemistry.** Strong and entire membrane expression of E-cadherin was seen in normal bronchial epithelium. Membrane expression of E-cadherin was decreased in cancer cells. N-cadherin expression was commonly absent in normal bronchial epithelium. Diffuse cytoplasmic expression of N-cadherin was detected in cancer cells. Strong cytoplasmic Btbd7 expression was accompanied by reduced membrane E-cadherin expression and accumulation of N-cadherin in cytoplasm in cancer cells (×400).

We examined E-cadherin and N-cadherin expression in NSCLC and non-tumor lung portions using IHC and analyzed their association with Btbd7 expression. The immunostaning pattern of E-cadherin and N-cadherin is shown in Figure 3. Normal epithelial cells usually show entire membrane staining of E-cadherin, while the immunostaining was decreased in cancer cells. Abnormal E-cadherin expression was seen in 64.6% (51/79) of NSCLC. N-cadherin was commonly absent in normal epithelial cells. N-cadherin expression was detected in cancer cells, usually exhibiting weak to moderate diffuse cytoplasmic staining. Abnormal cytoplasmic expression of N-cadherin was seen in 42.9% (27/63) of NSCLC. Relationship between Btbd7 expression and E-cadherin and N-cadherin status in NSCLC was shown in Table 2. Btbd7 expression was significantly associated with reduced membrane E-cadherin expression and increased cytoplasmic expression of N-cadherin in NSCLC (P < 0.05) (Table 2, Figure 3).

**Table 2:** **Correlation between Btbd7 expression and abnormal E-cadherin and N-cadherin expression in non-small cell lung cancer**

	Btbd7 expression		Correlation coefficient (r _s_)	*p*
	Negative	Positive		
E-cadherin				
Abnormal	16	35	0.274	0.011
Normal	17	11		
N-cadherin				
Abnormal	6	21	0.257	0.042
Normal	17	19		

*p and r_s_ (correlation coefficient) values were obtained by the Spearman correlation test.

### Evaluation of Btbd7 as a potential prognostic marker for NSCLC

**Figure 4:**
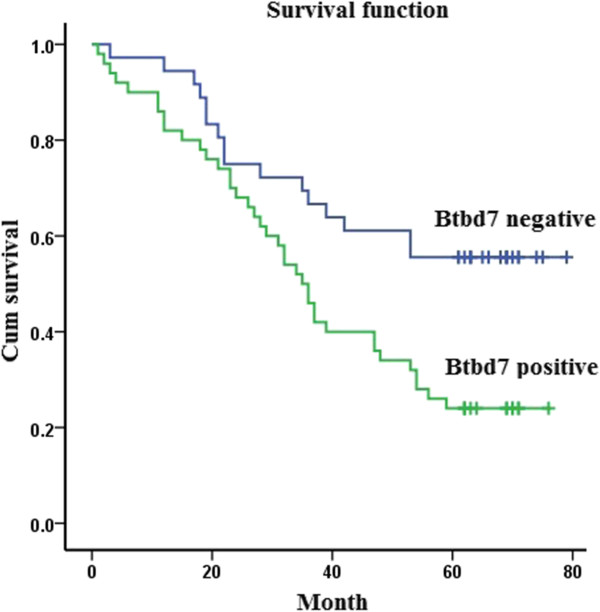
**The overall Kaplan-Meier survival curves show that the survival time of patients with Btbd7 expression (39.9 ± 3.5 m) was significantly shorter than that without Btbd7 expression (56.1 ± 4.5 m) (Log rank analysis,** ***p*** **< 0.05).**

We analyzed association between Btbd7 expression and the survival time of 86 patients with NSCLC. The overall mean suvival time was 47.1 (±3.0) m. The overall Kaplan-Meier survival curves reveal correlation between Btbd7 expression in NSCLC and shorter survival time of patients (*p* < 0.05). The survival time of patients with Btbd7 expression (39.9 ± 3.5 m) was significantly shorter than that without Btbd7 expression (56.1 ± 4.5 m) (p < 0.05) (Figure 4). Multivariate analysis shows that lymph node metastasis, TNM stage and Btbd7 expression are independent prognostic markers for NSCLC (*p* < 0.05) (Table 3).

**Table 3:** **Multivariate Cox proportional hazard analysis for overall survival of 86 patients with non-small cell lung cancer**

	B	SE	Wald	df	Sig.	Exp(B)
Lymph node metastasis	.885	.339	6.793	1	**.009**	2.422
TNM stage	.925	.344	7.247	1	**.007**	2.522
Grade	-.479	.345	1.929	1	.165	.620
Btbd7 expression	.719	.323	4.961	1	**.026**	2.052
Age	-.131	.303	.187	1	.665	.877
Gender	-.200	.306	.430	1	.512	.818

The bold numbers were considered statistically significant.

### Downregulation of Btbd7 restores membrane E-cadherin expression and inhibits lung cancer cell invasion in vitro

**Figure 5:**
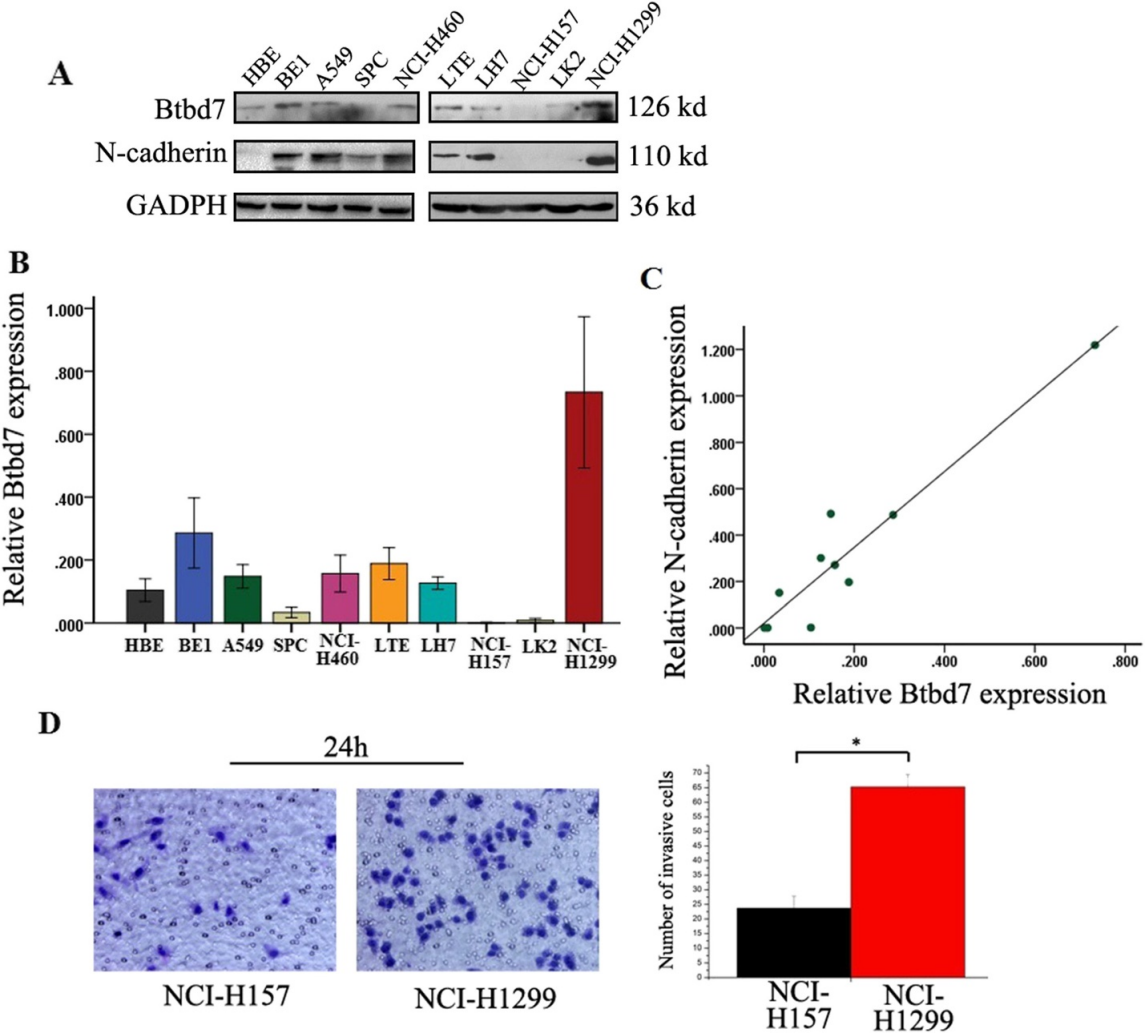
**Detection of Btbd7 expression in lung cancer and bronchial epithelial cell lines.** Higher expression level was detected in NCI-H1299 cells compared to other cancer cell lines and bronchial epithelial cell HBE **(A, B)**; N-cadherin expression was positively associated with Btbd7 expression **(A, C)**. Relative higher invasive ability (Transwell study, **D**) was detected in NCI-H1299 cells compared to NCI-H157 cells with relative lower Btbd7 expression.

**Figure 6:**
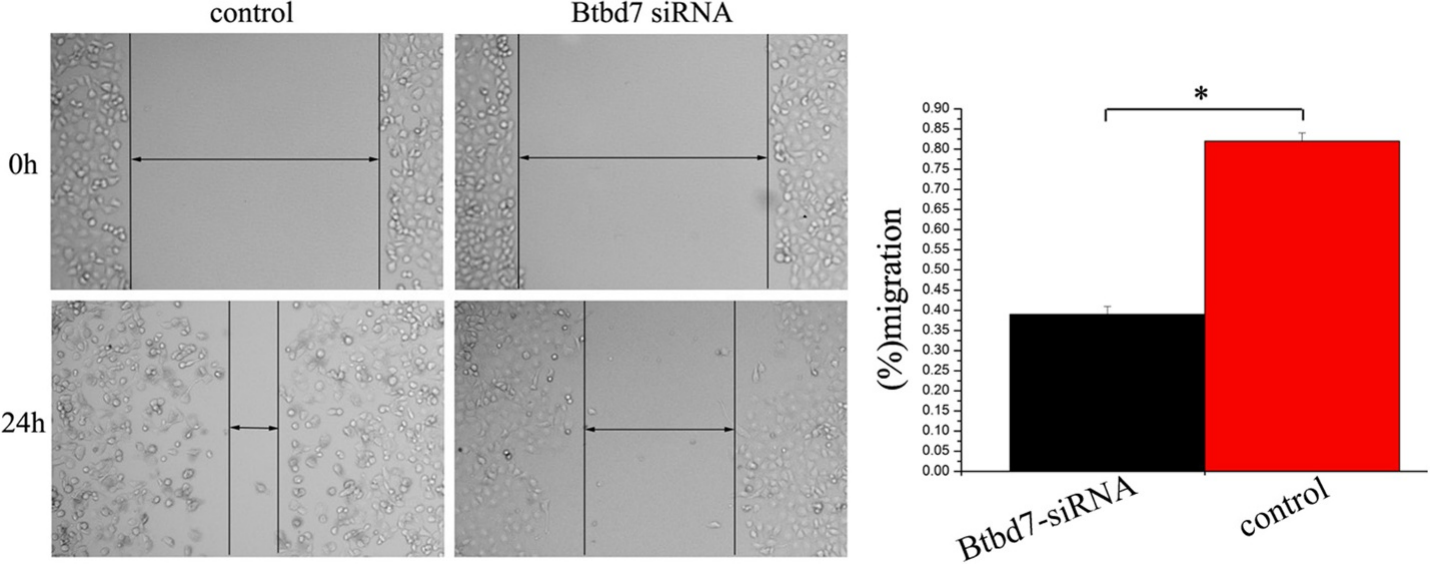
Scratch wounding assay shows downregulation of Btbd7 expression in NCI-H1299 cells using Btbd7 siRNA significantly inhibits cancer cell migration (* ***p*** **< 0.05).**

**Figure 7:**
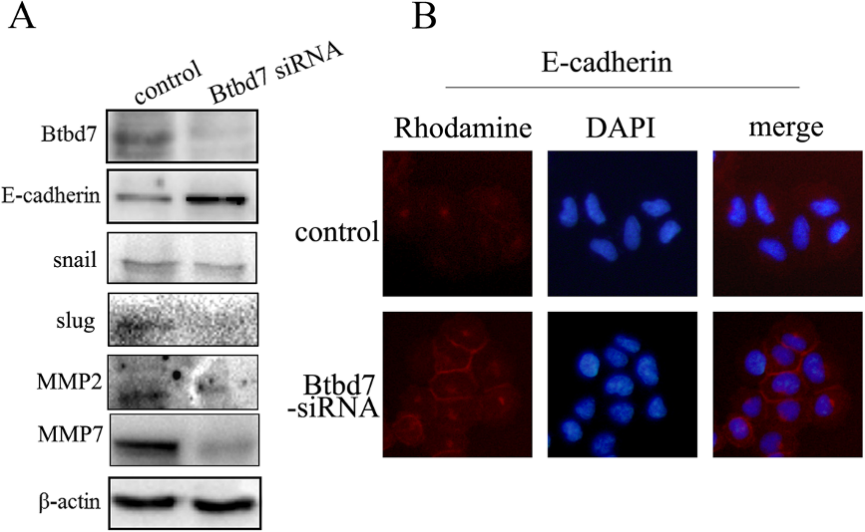
**Downregulation of Btbd7 expression in NCI-H1299 cells using Btbd7 siRNA significantly upregulates E-cadherin expression. (A)** Western blotting study shows effective downregulation of Btbd7 using Btbd7 siRNA significantly upregulates E-cadherin expression and downregulates slug, MMP2 and MMP7 expression (*p* < 0.05). **(B)** Immunofluorescent staining shows that downregulation of Btbd7 in NCI-H1299 cells effectively restores E-cadherin expression in cell membrane (×400).

We examined Btbd7 and N-cadherin expression in bronchial epithelial HBE cell and lung cancer cell lines including A549, BE1, LK2, NCI-H157, NCI-H460, NCI-H1299, LH7, LTE and SPC using Western blotting (Figure 5, A, B). Expression of Btbd7 was detected in these cells with different levels. Relative higher Btbd7 expression was detected in NCI-H1299 cells with strong expression of N-cadherin (Figure 5, A, B). Btbd7 and N-cadherin expression was positively related in these cell lines (r = 0.095, *p* < 0.001) (Figure 5, A, C). Transwell study shows higher invasive properties in NCI-H1299 cells than those in NCI-H157 cells with relative lower Btbd7 expression (Figure 5, A, B, D). We used NCI-H1299 cells with higher level of Btbd7 expression to perform in vitro study to investigate the function of Btbd7 in lung cancer cells. We transfected Btbd7 siRNA to downregulate Btbd7 expression in NCI-H1299 cells. The scratch wounding assay shows that downregulation of Btbd7 using Btbd7 siRNA significantly inhibits the migration ability of the cells (Figure 6). Western blotting study shows that downregulation of Btbd7 in NCI-H1299 cells significantly upregulated E-cadherin expression and downregulated snail 2 (slug), MMP2 and MMP7 expression in cancer cells (Figure 7, A). Immunofluorescent staining shows effective restoration of membrane expression of E-cadherin in NCI-H1299 cells after downregulation of Btbd7 (Figure 7, B).

## Discussion

Cancer relapse caused by cancer cell invasion and metastasis is the leading cause of death in patients with malignant tumors. However, the mechanisms involved in cancer invasion and metastasis are not fully understood yet. E-cadherin is a member of the cadherin superfamily and plays important roles in the maintenance of intercellular adhesion of epithelial cells. It is a calcium dependent transmembrane protein and a key component of adherens junctions [20]. Reduced expression of E-cadherin and dysfunction of E-cadherin complex in malignant tumors have been reported and implicated to cancer progression [4, 5]. Thus it is important to fully understand the mechanism involved in abnormality of E-cadherin expression and its association with cancer invasion and metastasis for improving lung cancer therapy based on potential cancer markers.

Yamada et al. found Btbd7 plays an important role in the branching morphogenesis of salivary glands and lungs during development [15]. Many organs form by branching of epithelia through the formation of clefts and buds. Yamada et al’s study shows that highly focal expression of Btbd7 promotes cell propagation and regulates epithelial cell motility through downregulation of E-cadherin during cleft formation. Their further study shows that overexpression of Btbd7 in MDCK (Madin-Darby canine kidney) cells induced labile cell-cell adhesions and scattering of MDCK epithelial cell colonies to dispersing cell clusters in both two-dimensional (2D) cultures and 3D collagen gels. These data indicate that Btbd7 may function as a mediator of epithelial dynamics and organ branching. Btbd7 protein contains two putative BTB/POZ domains. The BTB domain is a protein-protein interaction motif that determines a unique tri-dimensional fold with a large interaction surface [16]. Some BTB-containing proteins are known to control cellular processes including actin dynamics and cell-cycle regulation [16]. However, expression and function of Btbd7 in malignant tumors including lung cancer are largely unknown so far. In the current study, we found that expression of Btbd7 at protein level was elevated in NSCLC tissues compared to normal lung tissues. The pathological analysis shows that overexpression of Btbd7 in non-small cell lung cancer was associated with lymph node metastasis and advanced TNM stages, suggesting that Btbd7 may be an important molecule to promote the malignant phenotype of lung cancer. However 43.1% of the cases of I + II stages were Btbd7 positive though the positive rate was lower than that of III + IV. We can not exclude the possibility of a role of Btbd7 in early stage of NSCLC. Actually there is a report about Btbd7 mRNA expression in hepatocarcinoma indicating a role of Btbd7 to promote cancer cell proliferation though mechanism involved was not mentioned and not clear. Here we analyzed the clinical outcome and found that patients with Btbd7 expression in NSCLC tissues have shorter survival time than those without Btbd7 expression. These data indicate that Btbd7 expression may contribute to patients’ poor clinical outcome of NSCLC and may be a potential cancer marker for this tumor.

Our immunohistochemistry study shows that Btbd7 expression was associated with reduced membrane expression of E-cadherin and abnormal cytoplasmic N-cadherin expression in NSCLC. This result may at least partly explain its association with lymph node metastasis in NSCLC. We used siRNA to downregulate Btbd7 expression in lung cancer cells in vitro to investigate its possible function. We found that downregulation of Btbd7 using Btbd7 siRNA significantly inhibits the migration ability of the cells examined by scratch wounding assay. This result indicates that Btbd7 may contribute to cancer cell invasion and is consistent with the results in vivo that indicate its association with lymph node metastasis and patients’ poor clinical outcome. Our further study shows that downregulation of Btbd7 in NCI-H1299 cells significantly upregulated E-cadherin expression in cancer cells. E-cadherin and N-cadherin are two epithelial-mesenchymal transition (EMT) markers in cancer cells [21]. These results suggest that Btbd7 may be involved in EMT of lung cancer cells through regulating cadherins. The data also indicate that Btbd7 may contribute to cancer development and patients’ poor clinical outcome through regulation of E-cadherin and cancer cell dynamics. However, how exactly Btbd7 functions in this malignant tumor still needs further investigation. It is important to reveal mechanism of abnormal E-cadherin expression for understanding the mechanism of invasion and metastasis of lung cancer cells and finding new targets for clinical treatment.

## Conclusion

In this study, our findings suggest that the elevated expression of Btbd7 was a common abnormality in NSCLC compared to non-tumor portion and could play a role in NSCLC development including lymph node metastasis and thus contribute to patients’ poor outcome. Our study shows that Btbd7 is required for cancer cell invasion. We further demonstrated that Btbd7 expression was required for lung cancer cell invasion through regulating E-cadherin. These results will provide experimental evidence for improving lung cancer therapy based on potential cancer markers. In conclusion, our study provides evidence that Btbd7 contributes to lung cancer cell invasion and metastasis through regulating E-cadherin expression and may be a promising cancer marker.

## Electronic supplementary material

Additional file 1: **Relevant clinical data from the patients included in the study.** (XLS 24 KB)
